# Supplementary material for: Investigation of the Relationship between Lean Muscle Mass and Erythropoietin Resistance in Maintenance Haemodialysis Patients: A Cross-Sectional Study
Source: Int J Environ Res Public Health. 2022 May 7;19(9):5704. doi: 10.3390/ijerph19095704 (PMC9100199; doi:10.3390/ijerph19095704)
Supplement: Supplementary file 1 [file ijerph-19-05704-s001.zip › ijerph-1669394-supplementary.pdf]

Supplementary Table S1. Predictors of haemoglobin levels < 10 g/dL.

Variables	Univariate			Multivariate					
				Model 1			Model 2		
	b	OR (95% CI)	*p*	b	OR (95% CI)	*p*	b	OR (95% CI)	*p*
Age	0.85	2.35 (0.90–6.14)	0.082						
Sex	−0.70	0.50 (0.19–1.29)	0.151						
Diabetes mellitus	−0.45	0.64 (0.24–1.69)	0.364						
Ferritin	1.63	5.08 (1.80–14.3)	0.002	0.01	1.01 (1.00–1.01)	0.004	1.46	4.29 (1.40–13.2)	0.011
MIS	1.83	6.22 (2.12–18.2)	0.001	0.32	1.38 (1.04–1.83)	0.025	1.44	4.21 (1.32–13.4)	0.015
BMI	−0.23	0.79 (0.31–2.02)	0.624						
Relative OH	1.10	3.00 (1.13–7.99)	0.028	0.06	1.06 (1.00–1.16)	0.206	0.80	2.22 (0.72–6.85)	0.166
LTI	0.56	1.76 (0.68–4.53)	0.243						
FTI	0.85	2.35 (0.90–6.14)	0.082						

BMI, body mass index; FTI, fat tissue index; LTI, lean tissue index; MIS, malnutrition-inflammation score; OH, overhydration. b, logistic regression coefficients; OR, Odds Ratio. Model 1, multivariate logistic regression using continuous variables; Model 2, multivariate logistic regression using groups divided by medium.
